# Incorporating walking or cycling into car journeys to and from work: The role of individual, workplace and environmental characteristics

**DOI:** 10.1016/j.ypmed.2013.01.014

**Published:** 2013-03

**Authors:** Jenna Panter, Carol Desousa, David Ogilvie

**Affiliations:** a Medical Research Council Epidemiology Unit, Addenbrookes Hospital, Cambridge, CB2 0QQ, UK; b UKCRC Centre for Diet and Activity Research (CEDAR), Box 296, Institute of Public Health, Forvie Site, Robinson Way, Cambridge, CB2 0SR, UK; c Medical Research Council Biostatistics Unit, Institute of Public Health, Forvie Site, Robinson Way, Cambridge, CB2 0SR, UK

**Keywords:** Walking, Health promotion, Health behaviour, Physical activity

## Abstract

**Objective:**

Small increases in walking or cycling for transport could contribute to population health improvement. We explore the individual, workplace and environmental characteristics associated with the incorporation of walking and cycling into car journeys.

**Methods:**

In 2009, participants from the Commuting and Health in Cambridge study (UK) reported transport modes used on the commute in the last week as well as individual, workplace and environmental characteristics. Logistic regression was used to assess the explanatory variables associated with incorporating walking or cycling into car commuting journeys.

**Results:**

31% of car commuters (n = 419, mean age 43.3 years, SD 0.3) regularly incorporated walking or cycling into their commute. Those without access to car parking at work (OR: 26.0, 95% CI:11.8 to 57.2) and who reported most supportive environments for walking and cycling en route to work (highest versus lowest tertile, OR: 2.7, 95% CI 1.4 to 5.5) were more likely to incorporate walking or cycling into their car journeys.

**Conclusions:**

Interventions that provide pleasant and convenient routes, limit or charge for workplace car parking and provide free off-site car parking may encourage car commuters to incorporate walking and cycling into car journeys. The effects of such interventions remain to be evaluated.

## Highlights

► Walking or cycling for transport could contribute to population health improvement. ► We explore why commuters incorporate walking and cycling into car journeys. ► Supportive environments and lack of workplace car parking were important contributors.

## Introduction

Promoting physical activity is a public health priority (Beaglehole et al., 2011). Encouraging walking or cycling for transport could benefit population health not only by increasing physical activity, which helps prevent disease and improve wellbeing, but also by reducing noise, air pollution and carbon dioxide emissions, which may mitigate future climate change (Das and Horton, 2012). Even small increases in walking and cycling could lead to health benefits (Jarrett et al., 2012). Predominantly cross-sectional studies have found that those who report walking or cycling to work are healthier and less likely to be overweight than those who do not (Hamer and Chida, 2008; Wen et al., 2006).

Promoting active lifestyles may require social and environmental changes beyond the health sector (British Medical Association, 2012; Morabia and Costanza, 2012) and transport policies are increasingly aimed at shifting travel from car use towards walking and cycling (Department for Transport, 2011a). However, this may not be possible for everyone, particularly those who live far from work for whom it may be impractical to walk or cycle all the way (Iacono et al., 2008; Ogilvie et al., 2010). For example, commuters in the US and the UK travel 12.2 and 8.6 miles each way to and from work on average (Department for Transport, 2011b; Santos et al., 2010). It is possible to combine active and sedentary modes of travel by walking or cycling sections of a journey made mostly by car.

Behavioural epidemiological research on the correlates of walking and cycling has generally produced mixed evidence of associations (Panter and Jones, 2010), and although studies in the transport literature have also explored the factors associated with cycling (Heinen et al., 2010) and cycling in combination with public transport (Martens, 2007) we are unaware of any studies that have used disaggregated data on modes of commuter travel to assess the correlates of walking or cycling when used in combination with the car. Given the potential contribution of walking and cycling journeys to overall physical activity, understanding why people choose to make these journeys may help shape the design of intervention strategies to promote incidental physical activity. The aim of this study, therefore, was to examine the correlates of the incorporation of walking or cycling into commuting journeys made primarily by car.

## Methods

### Study design and sample

We examined cross-sectional data from the Commuting and Health in Cambridge study in Cambridge, UK, which is described in detail in a study protocol (Ogilvie et al., 2010) and baseline paper (Panter et al., 2011). Briefly, adults over the age of 16 working in Cambridge and living within 30 km of the city were predominantly recruited through workplaces (Yang et al., 2012) and sent postal questionnaires, a copy of which has been published elsewhere (Panter et al., 2011). Ethical approval was obtained from the Hertfordshire Research Ethics Committee and written informed consent was provided by each participant.

### Outcome: Travel modes used on the journey to and from work

In the absence of a valid measure of travel behaviour, we adopted an existing instrument shown to have acceptable test–retest reliability (Shannon et al., 2006) to assess all travel modes used on each journey to and from work in the last seven days. Participants were classified according to the most frequently reported travel mode (or combinations of modes) over the seven days. Where two or more modes or combinations of modes were reported equally frequently (n = 46), we made the conservative assumption of assigning participants to the least active category.

To identify those walking a substantive part of their journey, we considered participants' responses to a question which assessed the typical duration of the walking stage of their commute. We reclassified those individuals who reported walking for less than 5 minutes as using the ‘car only’ (n = 5). Repeating the analysis with and without these cases made no material difference to the results.

### Exposures

#### Individual and household characteristics

Participants reported their date of birth, gender, educational qualifications, possession of a driving licence, difficulty walking, height, weight, housing tenure, household composition, access to cars and bicycles within the household and physical activity at work. Body mass index (BMI) was calculated and weight status was assigned based on internationally recognised cutoffs (World Health Organisation, 2000). Using a Geographical Information System (ArcGIS v9.0), urban–rural status (Bibby and Shepherd, 2004) was assigned using the Census Output Area of the home postcode.

#### Workplace-related characteristics

Participants also reported characteristics of their workplace: car parking availability, distance between home and work, and the postcode or its location in Cambridge. We hypothesised that those car users who commuted into the heart of the city might be more likely to incorporate walking and cycling to avoid traffic congestion in the inner city. To capture the context of their commute we created a variable based on home (in Cambridge itself, or in the surrounding towns, villages and rural areas) and workplace locations (in the heart of the city, or on the outskirts).

#### Perceived environment on the route between home and work

Participants reported their level of agreement with seven statements that could describe the environment along their route to and from work using a five-point Likert scale (for example: ‘it is pleasant to walk’) (Panter et al., 2011). A total score was generated, whereby higher scores represented a more supportive perceived route environment for walking and cycling, and divided approximately into tertiles. If one or two items were missing, the missing responses were conservatively imputed by replacing them with the response that was least likely to be associated with walking or cycling based on the literature (Panter and Jones, 2010), otherwise the composite score was coded as missing.

#### Psychological measures relating to car use

Respondents reported their agreement with eight statements using a five-point Likert scale from a previously validated questionnaire (Hardeman et al., 2009) assessing the constructs of perceived behavioural control (PBC), intention, attitudes and subjective norms applied to car use. Mean scores were computed and classified as either above and below the median. If one item within a pair was missing, the score was coded to missing. Habit strength for using the car for the commute was assessed using participants' reported agreement with seven statements derived from the Habit Strength Index (Verplanken and Orbell, 2003) using a five-point Likert scale. Mean scores and imputed missing responses were computed using the rules described above. As the distribution of scores (range 1–5) was positively skewed, a binary summary variable was created (0: those with a mean score = 1 and 1: a mean score > 1).

### Statistical analyses

Before models were fitted, descriptive data were summarised using percentages and t-tests, chi-squared and Mann–Whitney tests. Univariate associations were examined using logistic regression models, with those reporting ‘car only’ as their most frequent travel mode over the last seven days as the reference category. Using explanatory variables for which a significance of p < 0.25 was obtained in univariate analysis (Hosmer and Lemeshow, 1989), multivariable regression models were built up in stages to ensure transparency and to assess the contributions of sets of variables one at a time. Our hierarchical approach allowed us to explore the possibility of mediating mechanisms, which has been suggested as an important area for research (Kremers et al., 2006). We hypothesised that any effect of favourable perceptions of the route environment on walking and cycling behaviours might be directly mediated by weak psychological measures relating to car use (Kremers et al., 2006).

We also tested for any moderating effects of habit strength and availability of car parking at work on the association between perceptions of the environment and behaviour in the maximally adjusted model, as previous conceptual models postulated that habit may act in this way (Kremers et al., 2006) and car parking availability appears to be strongly associated with travel behaviour (Willson and Shoup, 1990). Analyses were not adjusted for workplace clustering because the intraclass correlation of behaviour (ICC) within workplaces was 0. All analyses were conducted in Stata 11.1.

## Results

### Study sample

Of 1582 participants who were sent a questionnaire, 1142 returned a completed questionnaire and reported commuting in the last seven days. In total, 419 participants who reported using a car most often on their commute were included in this analysis (Table 1), of whom 31% (n = 131) reported regularly incorporating walking or cycling into their car journeys. These participants were more likely to be older (mean age 43.7 versus 41.4 years), female (76.6% versus 63.9%) and overweight (43.7% versus 33.5%), to live in a rural location (55.9% versus 21.4%) and to own their home (85.4% versus 65.2%) than those excluded (all p < 0.01). Participants included were also more likely to have one or more children in their household (32.7% versus 21.4%, p = 0.07) and to have a longer journey between home and work (mean 20.4 km versus 8.2 km, p = 0.001) than those excluded from analysis.

### Explanatory variables associated with incorporation of active modes into car journeys

In unadjusted analyses, those with a standing or manual occupation, who lived in a less deprived area, who reported having no access to car parking at work, who reported a supportive route environment and who reported positive psychological measures relating to car use were more likely to incorporate walking or cycling into a car journey (Table 2). Those who were overweight or obese were less likely to do so.

In multivariable regression models, relatively few explanatory variables were associated with the likelihood of incorporating walking or cycling into car journeys (Table 3). In maximally adjusted models, only those who reported having to pay for parking (OR: 4.1, 95% CI 2.2 to 7.5) or had no car parking at work (OR: 26.0, 95% CI 11.8 to 57.2), and those who reported the most supportive environment for walking and cycling on their route (highest versus lowest tertile, OR: 2.7, 95% CI 1.4 to 5.5) were significantly more likely to incorporate walking or cycling into their car commutes. Neither habit (p = 0.205) nor the availability of workplace car parking (p = 0.532) showed moderating effects on the association between perceptions of the route environment and behaviour.

## Discussion

### Principal findings

In this study of healthy working adults, we found that only two of the potential explanatory variables remained significant in the maximally adjusted model: those who reported having to pay for or having no car parking at work, and those who report having a supportive environment for walking and cycling en route, were more likely to incorporate walking or cycling into car commuting journeys.

### Strengths and limitations

As far as we are aware, this is one of the first studies to explore why car commuters integrate walking and cycling into their journeys. The findings may therefore help inform the development of interventions in areas where car commuting is prevalent and where substituting walking and cycling for the entire journey may not always be practical. Second, a broad range of potential explanatory variables were included and given the dearth of literature on this topic, these analyses make an important and novel contribution. Participants who reported using active modes in combination with the car reported an average of 12 minute walking and 17 minute cycling per day on their commutes. Understanding the reasons for engaging in such behaviours may therefore have important implications for population physical activity promotion. However, these analyses were cross-sectional in nature and therefore no causal associations can be inferred. Using the last seven days as the frame of reference allowed detailed information on travel behaviour to be collected, but may also have captured short-term fluctuations. However, as most participants' seven-day travel patterns were similar to those in the last four weeks (reported elsewhere in the questionnaire), seven-day reported behaviour appears to be typical. Our sample comprised individuals living in and around Cambridge, which is known for its cycling culture (Office of National Statistics, 2012). Our findings may therefore not be generalizable to other contexts where cycling, in particular, is less prevalent. Furthermore, our psychological measures were framed in terms of car commuting rather than walking or cycling and participants reported the characteristics and conditions on or along their entire route to work; we did not examine the influence of objectively-measured characteristics of the route.

### Importance of workplace-related characteristics

The explanatory variable most strongly associated with the target behaviour was the availability of car parking at work; those who reported having no car parking at work were much more likely to incorporate walking and cycling into their car commute. Previous studies have suggested that subsidised workplace car parking is a major influence on regular car commuting (Willson and Shoup, 1990) and inversely associated with cycling (Buehler, 2012). Qualitative investigations in a sample of our participants provide insight into how walking and cycling were combined with car use (Guell et al., 2012; Jones and Ogilvie, 2012). Some reported parking for free on streets within walking distance of their workplace, whilst others used park-and-ride sites creatively. Rather than using the latter to access dedicated bus services as originally intended, they used these as free car parks and continued their journeys on foot or by bike. These observations suggest that limiting or charging for on-site workplace parking and simultaneously providing free off-site car parking, may encourage walking or cycling as part of longer car journeys. However, the effects of such policies on walking, cycling or overall levels of physical activity are unknown and would require careful evaluation.

Previous research has consistently shown that distance between home and work is an important correlate of behaviour in that those who live closer to work are more likely to walk or cycle (Panter and Jones, 2010). In contrast, we found that distance was not associated with the likelihood of incorporating walking and cycling into car journeys, suggesting that even those with long commutes were able to integrate incidental physical activity into their commute. Interventions involving public transport or park-and-ride facilities may have particular potential to benefit population groups that are sometimes neglected in strategies to promote active commuting, such as commuters living in rural areas who typically have longer journeys to work than their urban counterparts.

### Importance of perceptions of the route environment

We also found that those participants who reported a supportive route for walking and cycling were more likely to incorporate walking and cycling into their car journeys. This association persisted after adjustment for attitudes, social norms and habits. We cannot say whether these individuals chose to walk and cycle because they perceived their routes to be more supportive of walking or cycling, or perceived their routes as safe and convenient because of their awareness and repeated exposure. Analyses exploring associations between perceived and objective measures of the environment tend to show weak associations between them (Ball et al., 2008; Hoehner et al., 2005). More research is required to understand how actual environmental conditions are perceived and if these perceptions differ between population groups, given that creating a supportive environment may be a ‘necessary but not sufficient’(Giles-Corti and Donovan, 2002) component of a wider intervention strategy to promote walking and cycling at a population level.

## Conflict of interest statement

The authors declare there is no conflict of interest.

## Figures and Tables

**Table 1:** Descriptive characteristics of the sample from Cambridge, UK.

Variable	Percentage (number)			
All participants (n = 419)	Car (n = 288)	Car in combination with walking or cycling (n = 131)	p
*Personal characteristics*				
Mean age in years (SD)	43.7 (11.9)	43.8 (10.8)	43.5 (11.8)	0.816
Gender				
Male	23.4 (98)	24.0 (69)	22.1 (29)	0.683
Female	76.6 (321)	76.0 (219)	77.9 (102)	
Weight status				
Underweight/normal	56.3 (232)	53.4 (151)	62.8 (81)	0.073
Overweight/obese	43.7 (180)	46.6 (132)	37.2 (48)	
Work type				
Sedentary/standing	81.8 (342)	83.6 (240)	77.9 (102)	0.157
Manual	18.1 (76)	16.4 (47)	22.1 (29)	
Difficulty walking				
Yes	2.4 (10)	2.1 (6)	3.0 (4)	0.546
No	97.6 (409)	97.9 (282)	97.0 (127)	
Number of children in the household				
None	67.3 (282)	67.3 (191)	71.0 (91)	0.525
One or more	32.7 (137)	32.3 (97)	29.3 (40)	
Urban–rural status				
Urban	44.3 (185)	42.9 (123)	46.6 (61)	0.479
Rural	55.9 (234)	57.1 (164)	53.4 (70)	

*Socio-economic characteristics*				
Highest educational qualifications				
Lower than degree	35.1 (146)	35.3 (101)	34.6 (45)	0.890
Degree or equivalent	64.9 (270)	64.7 (185)	65.4 (85)	
Housing tenure				
Owned	85.4 (356)	84.6 (242)	87.0 (114)	0.518
Privately rented/shared ownership/social housing	14.6 (61)	15.4 (44)	13.0 (17)	
Index of multiple deprivation				
Quartile 1 (most deprived)	291 (25.0)	28.5 (82)	17.5 (23)	0.093
Quartile 2	291 (25.0)	22.9 (66)	29.7 (39)	
Quartile 3	291 (25.0)	25.4 (73)	25.9 (34)	
Quartile 4 (least deprived)	290 (25.0)	23.2 (67)	26.7 (35)	

*Workplace-related characteristics*				
Distance to work				
< 10 km	22.9 (97)	21.8 (63)	26.0 (34)	0.642
10.01–19.99 km	27.0 (112)	26.8 (77)	26.8 (35)	
20 km and over	50.1 (210)	51.4 (148)	47.2 (62)	
Workplace car parking				
Free parking	48.5 (203)	62.2 (179)	18.3 (24)	0.001
Pay for parking	35.3 (148)	32.6 (94)	41.2 (54)	
No parking	16.2 (68)	5.2 (15)	40.5 (53)	
Geographical context of commuting journey				
Commuting to the heart from within the city	24.7 (103)	26.7 (76)	20.6 (27)	0.298
Commuting to the outskirts from within the city	26.9 (112)	28.0 (80)	24.4 (32)	
Commuting to the heart from outside the city	22.6 (94)	20.7 (59)	26.7 (35)	
Commuting to the outskirts from outside the city	25.7 (107)	24.6 (70)	28.3 (37)	

*Perceptions of the route environment*^a^				
Reported the least supportive route (lowest tertile)	33.4 (138)	39.4 (111)	20.6 (27)	0.001
Middle tertile	34.2 (158)	37.9 (107)	38.9 (51)	
Reported the most supportive route (highest tertile)	28.3 (122)	22.7 (64)	40.5 (53)	

*Psychological measures relating to car use*				
Intention to use car (2 items)				
Below median	56.4 (234)	61.4 (175)	45.4 (59)	0.002
Above median	43.6 (181)	38.6 (110)	54.6 (71)	
Positive attitude towards car (2 items)				
Below median	51.9 (214)	59.7 (169)	34.8 (45)	0.001
Above median	48.1 (198)	40.3 (114)	65.1 (84)	
Perceived behavioural control (2 items)				
Below median	57.4 (236)	63.3 (179)	44.6 (57)	0.008
Above median	42.6 (175)	36.7 (104)	55.4 (71)	
Social norm (2 items)				
Below median	59.0 (242)	66.7 (188)	42.2 (54)	0.001
Above median	41.0 (168)	33.3 (94)	57.8 (74)	
Habit strength				
Low habit strength	50.5 (210)	54.5 (157)	40.5 (53)	0.008
High habit strength	49.5 (206)	45.4 (131)	59.6 (78)	

*Physical activity*				
Mean minutes/day spent walking on the commute (SD)	5.14 (11.9)	0.65 (3.1)	11.8 (14.7)	0.001
Mean minutes/day spent cycling on the commute (SD)	4.3 (9.6)	1.3 (5.1)	17.4 (18.2)	0.001

Percentages represent column percentages. Data collected in 2009 in Cambridge, UK. p values represent differences between ‘car only’ and ‘car in combination with walking or cycling’ groups.

a The seven items comprising the perceptions of the route environment were: ‘It is pleasant to walk’, ‘There is convenient public transport’, ‘There is little traffic’, ‘There are no convenient routes for walking’, ‘It is safe to cross the road’,’ The roads are dangerous for cyclists’ and ‘There are convenient routes for cycling’. Further details can be found in Panter et al. (2011).

**Table 2:** Unadjusted models for odds of incorporating walking or cycling into car journeys.

Variable	OR (95% CI)	p
*Personal characteristics*		
Age (under 30)		
30–49	0.79 (0.41, 1.54)	0.926
50 +	0.92 (0.45, 1.85)	
Gender (reference: male)		
Female	1.11 (0.68, 1.81)	0.683
Weight status (reference: underweight or normal weight)		
Overweight or obese	0.68 (0.44, 1.04)	0.074
Work type (reference: sedentary)		
Standing/manual	1.45 (0.87, 2.44)	0.158
Difficulty walking (reference: no)		
Yes	0.67 (0.18, 2.43)	0.549
Children (reference: no children)		
At least 1 child in the household	0.87 (0.55, 1.35)	0.525
Urban–rural status (reference: urban)		
Rural	0.86 (0.57, 1.30)	0.479

*Socio-economic characteristics*		
Highest educational status (reference: less than degree)		
Degree	0.97 (0.63, 1.50)	0.890
Housing tenure (reference: owns their home)		
Privately rented/shared ownership/social housing	0.82 (0.45, 1.50)	0.519
Index of multiple deprivation (reference: most deprived quartile)		
Quartile 2	2.10 (1.15, 3.87)	0.117
Quartile 3	1.66 (0.90, 3.07)	
Quartile 4 (least deprived)	1.86 (1.00, 3.45)	

*Workplace-related characteristics*		
Distance to work (reference: < 10 km)		
10.01–19.99 km	0.84 (0.47, 1.50)	0.342
20 km and over	0.77 (0.47, 1.29)	
Workplace car parking (reference: free parking)		
Pay for parking	4.28 (2.49, 7.37)	0.001
No parking	26.4 (12.90, 53.83)	
Geographical context (reference: commuting to the heart from within the city)		
Commuting to the outskirts from within the city	0.40 (0.13, 1.23)	0.111
Commuting to the heart from outside the city	0.61 (0.22, 1.69)	0.348
Commuting to the outskirts from outside the city	0.45 (0.16, 1.23)	0.121

*Perceptions of the route environment*		
Sum of perceived environment of route (reference: least supportive route (lowest tertile))		
Middle tertile	1.95 (1.15, 3.35)	0.001
Reported the most supportive route (highest tertile)	3.04 (1.95, 5.94)	

*Psychological measures relating to car use*		
Mean intention to use car (reference: low)		
High	1.91 (1.25, 2.91)	0.002
Mean attitude towards car (reference: low)		
High	2.76 (1.79, 4.26)	0.001
Mean social norm (reference: low)		
High	2.74 (1.78, 4.21)	0.001
Mean PBC (reference: low)		
High	2.14 (1.40, 3.27)	0.001
Habit strength (reference: low)		
High	1.73 (1.13, 2.64)	0.011

OR: Odds Ratio; CI: Confidence intervals; PBC: Perceived behavioural control. Where one p-value is reported for several categories, it refers to a test for trend across the groups. Data collected in 2009 in Cambridge, UK.

**Table 3:** Multivariable regression models for odds of incorporating walking and cycling into car journeys.

Variables	Model 1		Model 2		Model 3		Model 4		Model 5	
OR (95% CI)	p	OR (95% CI)	p	OR (95% CI)	p	OR (95% CI)	p	OR (95% CI)	p
*Personal characteristics*										
Overweight or obese (reference: underweight or normal)	0.70 (0.45, 1.08)	0.108	0.69 (0.41, 1.14)	0.149	0.64 (0.39, 1.07)	0.091	0.72 (0.42, 1.22)	0.216	0.71 (0.42, 1.21)	0.203
Standing or manual work (reference: sedentary work)	1.43 (0.84, 2.45)	0.192	1.15 (0.62, 2.13)	0.666	1.34 (0.71, 2.52)	0.366	1.40 (0.73, 2.69)	0.308	1.41 (0.73, 2.70)	0.305

*Socio-economic characteristics*										
Index of multiple deprivation (reference: most deprived quartile)										
Quartile 2	1.94 (1.03, 3.63)	0.100	1.80 (0.87, 3.72)	0.184	1.83 (0.88, 3.83)	0.346	2.14 (0.99, 4.65)	0.407	2.09 (0.96, 4.57)	0.431
Quartile 3	1.65 (0.88, 3.11)		1.57 (0.75, 3.30)		1.64 (0.78, 3.45)		1.77 (0.82, 3.83)		1.74 (0.80, 3.78)	
Quartile 4 (least deprived)	1.88 (1.00, 3.56)		1.81 (0.87, 3.79)		1.56 (0.75, 3.27)		1.57 (0.73, 3.36)		1.55 (0.72, 3.32)	

*Workplace-related characteristics*										
Workplace car parking (reference: free parking)										
Pay for parking			4.20 (2.34, 7.56)	0.001	3.94 (2.18, 7.09)	0.001	4.07 (2.20, 7.50)	0.001	4.07 (2.20, 7.50)	0.001
No parking			27.93 (13.14, 59.37)		27.25 (12.69, 58.53)		25.97 (11.79, 57.24)		25.96 (11.78, 57.19)	

*Perceptions of the route environment*										
Sum of perceived environment of route (reference: reported the least supportive route (lowest tertile))										
Middle tertile					1.79 (0.95, 3.35)	0.002	1.77 (0.91, 3.44)	0.006	1.79 (0.92, 3.51)	0.007
Reported the most supportive route (highest tertile)					2.77 (1.44, 5.34)		2.68 (1.33, 5.39)		2.74 (1.35, 5.54)	

*Psychological measures relating to car use*										
Positive attitude towards car (reference: negative)							1.75 (0.94, 3.26)	0.077	1.82 (0.96, 3.44)	0.068
Strong social norm (reference: low)							1.57 (0.83, 3.00)	0.168	1.65 (0.84, 3.23)	0.146
Strong habit (reference: low)									0.86 (0.47, 1.60)	0.638

OR: Odds Ratio; CI: Confidence interval. Where one p-value is reported for several categories, it refers to a test for trend across the groups. Data collected in 2009 in Cambridge, UK. Model 1: personal and socio-economic characteristics; Model 2: personal, socio-economic and workplace-related characteristics; Model 3: personal, socio-economic and workplace-related characteristics and perceptions of the route environment; Model 4: personal, socio-economic and workplace-related characteristics, perceptions of the route environment and psychological measures relating to car use; Model 5: personal, socio-economic and workplace-related characteristics, perceptions of the route environment, psychological measures relating to car use and habit strength for car use.
